# Hospitalization Trends and Outcomes of Eosinophilic Esophagitis in the United States: A Decade-Long Nationwide Analysis

**DOI:** 10.7759/cureus.44315

**Published:** 2023-08-29

**Authors:** Robert Kwei-Nsoro, Pius E Ojemolon, Bashar Attar, Hafeez Shaka, Patricia Zarza-Gulino, Michael Asare, Eugene N Annor, Benjamin Mba

**Affiliations:** 1 Internal Medicine, John H. Stroger, Jr. Hospital of Cook County, Chicago, USA; 2 Gastroenterology and Hepatology, RUSH (Rush University System for Health) University Medical Center, Chicago, USA; 3 Gastroenterology and Hepatology, John H. Stroger, Jr. Hospital of Cook County, Chicago, USA; 4 Internal Medicine, BronxCare Hospital Center, New York, USA; 5 Internal Medicine, University of Illinois Chicago, Chicago, USA; 6 Internal Medicine, Yale New Haven Hospital, New Haven, USA

**Keywords:** national inpatient sample, trends analysis, hospitalizations, esophageal diseases, eosinophilic esophagitis

## Abstract

Background

Eosinophilic esophagitis (EoE) is a chronic antigen-mediated esophageal disease characterized by infiltration of the esophageal mucosa by eosinophils. The prevalence of EoE continues to rise worldwide. However, certain aspects of the epidemiology and pathogenesis remain unclear.

Methods

This study examined the hospitalization trends of EoE using an extensive inpatient database in the United States, the National (Nationwide) Inpatient Sample (NIS), to identify hospitalizations between 2010 and 2019. We assessed patient demographics as well as hospital-specific variables using the NIS. We obtained the prevalence rate of EoE for each year and used joinpoint regression analysis to obtain trends after adjusting the rate for age and gender. We also sought to characterize the outcomes of these hospitalizations by obtaining the mortality rate, length of stay (LOS), and total hospital charges (THC).

Results

Of 305 million hospitalizations included in the study, 33,878 were for EoE. The prevalence rate per 100,000 hospitalizations of EoE increased from 6.6 in 2010 to 15.5 in 2019. The annual percentage change obtained from the joinpoint regression analysis was 13.3% from 2010 to 2014 and 7.2% from 2014 to 2019. Most of the hospitalizations were among the male gender and young adults. Almost 95% of hospitalizations across the study period were seen in urban hospitals. We did not notice any significant trend in the mortality rates or length of stay over the study period. The THC increased significantly across the study period.

Conclusion

There has been an upward trend in the average prevalence rate of EoE over the decade from 2010 to 2019 which almost parallels that of inflammatory bowel disease. This represents a significant burden of disease for a condition that was initially recognized in the late 20th century.

## Introduction

Eosinophilic esophagitis (EoE) is a chronic antigen-mediated esophageal disease characterized clinically by dysphagia and food impaction and histologically by eosinophil infiltration of the esophageal mucosa [1]. The incidence of EoE continues to rise worldwide and has evolved into a distinct clinical entity over the years after initial recognition in the late 20th century [2].

The increased use of endoscopies and biopsies may account for some part of the rising EoE incidence but can not explain the meteoric rise in EoE cases. EoE has been mainly described in urban areas compared to rural areas, which strongly suggests that environmental factors may be critical in disease pathogenesis [3].

Despite the rising incidence of EoE worldwide, some aspects of the epidemiology of EoE are poorly understood, particularly in the United States (US), given the relatively scant literature on the subject. We decided to use a large representative national database to provide insights into the hospitalization trends of EoE over the decade in the US subregion.

## Materials and methods

This was a decade-long retrospective longitudinal study involving hospitalizations with EoE in the US obtained from the National (Nationwide) Inpatient Sample (NIS) databases from 2010 to 2019. The NIS is a database of hospital inpatient stays derived from billing data from hospitals across the US covering more than 97% of the population and is weighted to obtain US national estimates [4,5]. The NIS lacks patient identifiers. In keeping with other studies involving HCUP databases, the NIS does not require Cook County Health Institutional Review Board approval for analysis.

We searched the NIS database for EoE hospitalizations using International Classification of Diseases (ICD) codes K20.0 and 530.13. We excluded hospitalizations with patients aged less than 18 years. We assessed patient demographics and hospital-specific variables from the variables included in the NIS. We also assessed the comorbidity burden using Sundararajan’s adaptation of the modified Deyo’s Charlson comorbidity index (CCI), which has been used in prior Healthcare Cost and Utilization Project (HCUP) database research [6,7]. We highlighted the biodemographic trends over time for hospitalizations with EoE. Specifically, we obtained the prevalence rate of EoE per 100,000 hospitalizations. We also sought to characterize the outcomes of these hospitalizations by obtaining the mortality rate, length of stay (LOS), and total hospital charges (THC).

Data were analyzed using Stata Statistical Software: Release 17 (2021; StataCorp LLC, College Station, Texas, US). Age was divided into three categories: 18 - 44 years for young adults, 45 - 64 years representing middle-aged adults, and 65 years and above for the elderly. The prevalence of EoE was calculated following the HCUP methodology for disease incidence and prevalence [8]. Multivariable regression analysis was used to estimate the prevalence rates adjusted for age categories, sex, and race using predictive margins. We then used joinpoint regression analysis to obtain the trends in prevalence rate over the study duration. This methodology has been adopted in prior HCUP database research [9-11].

## Results

Of 305 million hospitalizations over the decade, 33,878 were for EoE. The biodemographic characteristics of EoE hospitalizations are shown in Table 1. The prevalence rate per 100,000 hospitalizations of EoE increased from 6.6 to 15.5 over the decade studied.

**Table 1:** Biodemographic data of hospitalizations with eosinophilic esophagitis CCI: Charlson comorbidity index, EoE: eosinophilic esophagitis, MHOI: median household income national quartile for patient ZIP code, SD: standard deviation from the mean

Variable	2010	2011	2012	2013	2014	2015	2016	2017	2018	2019	p-value
Total hospitalizations, million, N = 305	31.4	31.5	30.7	30.0	29.8	30.2	30.2	30.4	30.3	30.2	
Total hospitalizations with EoE	2075	2386	2780	2904	3305	3695	3740	3879	4444	4670	
EoE prevalence rate per 100,000 hospitalizations	6.6	7.6	9.1	9.7	11.1	12.2	12.4	12.8	14.7	15.5	
Mean age ± SD, years	46.2 ± 18.1	47.5 ± 19.6	45.0 ± 18.3	47.8 ± 18.3	46.5 ± 17.8	46.4 ± 17.8	46.7 ± 18.7	46.9 ± 18.6	48.1 ± 18.3	47.9 ± 18.6	0.041
Age categories, %											0.238
Young adults (18-44)	49.4	46.6	52.5	45.4	48.0	47.9	48.5	47.4	44.8	47.1	
Middle-aged (45-64)	34.1	32.9	31.3	35.5	32.5	34.4	31.7	33.6	34.5	30.1	
Elderly (≥65)	16.5	20.5	16.2	19.1	19.5	17.7	19.8	19.0	20.7	22.8	
Female, %	44.2	42.1	48.7	49.2	45.3	46.8	52.9	46.7	46.9	48.9	0.032
Race, %											<0.001
White	73.9	77.2	78.2	75.2	75.6	73.8	76.3	78.6	78.6	80.8	
Black	4.7	6.5	9.0	7.8	7.0	9.8	7.9	8.6	7.8	7.4	
Hispanic	3.5	5.6	4.2	5.3	6.1	3.9	6.4	5.5	6.6	4.7	
Others	17.9	12.7	8.6	11.7	11.3	12.3	9.4	7.3	7.0	7.1	
CCI score, %											<0.001
0	49.1	46.6	49.1	49.1	48.6	43.8	39.6	40.1	41.3	40.8	
1	32.1	29.0	28.4	28.7	27.5	30.2	31.4	28.9	28.8	30.1	
2	10.3	13.3	10.4	11.5	11.8	11.0	11.8	14.7	13.7	13.3	
≥3	8.5	11.1	12.1	10.7	12.1	15.0	17.2	17.3	16.2	15.8	
Primary payer, %											<0.001
Medicare	24.0	26.6	22.5	26.5	23.3	26.3	25.2	24.7	28.0	28.8	
Medicaid	11.1	8.0	13.7	10.7	14.1	16.7	15.5	19.2	16.3	14.7	
Private insurance	57.8	57.6	55.0	56.3	57.1	52.3	54.6	52.1	50.1	51.6	
No insurance	7.1	7.8	8.9	6.5	5.5	4.7	4.7	4.0	4.8	4.9	
MHOI quartile, %											0.290
1	15.7	19.4	20.7	20.6	19.3	25.1	21.2	21.5	21.0	21.3	
2	27.7	20.7	25.5	25.8	25.0	24.0	22.7	21.9	25.3	23.5	
3	28.3	27.8	26.1	25.1	24.2	27.0	26.9	25.7	26.7	24.8	
4	28.3	32.1	27.7	28.5	31.5	23.9	29.2	30.9	27.0	30.4	
Hospital Bed-size, %											<0.001
Small	11.1	9.2	10.3	12.1	17.4	15.3	13.6	14.0	15.3	19.9	
Medium	20.3	25.3	25.2	22.9	27.2	28.4	27.3	27.2	26.8	26.6	
Large	68.6	65.5	64.5	65.0	55.4	56.3	59.1	58.8	57.9	53.5	
Hospital region, %											0.990
Northeast	15.6	20.7	20.1	17.7	18.8	18.0	17.7	19.6	19.7	18.6	
Midwest	28.6	29.7	29.9	26.5	27.4	29.9	27.0	26.4	26.4	28.3	
South	28.9	29.8	28.1	34.8	32.8	29.6	31.2	29.5	31.3	31.7	
West	26.9	19.8	21.9	21.0	22.5	22.5	22.6	24.5	22.6	21.4	
Location/teaching status of the hospital, %											<0.001
Rural	6.4	6.5	4.1	4.7	4.3	3.6	3.7	4.9	4.1	4.7	
Urban non-teaching	48.1	37.5	35.1	39.2	20.1	25.6	23.8	16.9	16.8	25.6	
Urban teaching	45.5	56.0	60.8	56.1	75.6	70.8	72.5	78.2	79.1	69.7	

Young adults made up the majority (47.8%) of EoE hospitalizations. The trend in age categories among EoE hospitalizations showed no significant change. The majority of EoE hospitalizations were among the male gender and White population. The proportion of patients with a CCI score of 0 showed a downward trend over the study period (from 49% in 2010 to 41% in 2019), while that of patients with a CCI score ≥3 showed an upward trend (from 8.5% in 2010 to 15.8% in 2019), with p <0.001. More than 50% of hospitalizations of EoE were hospitalizations with private insurance. There was an upward trend in the proportions of hospitalizations admitted to urban teaching hospitals over the period (45.5 in 2010 vs. 69.7 in 2019). The majority of the EoE hospitalizations were from the South and Midwest regions of the US.

Table 2 shows the outcomes of hospitalizations of EoE. Mortality among EoE hospitalizations showed an upward trend over the period, but the trend was not statistically significant (p = 0.605). The mean LOS of stay remained similar over the decade, with a mean LOS of 4.4 days. The total cost of hospitalization showed an upward trend across the study period.

**Table 2:** Outcomes of hospitalizations with eosinophilic esophagitis LOS: length of hospital stay, THC: total hospital charges

Variable	2010	2011	2012	2013	2014	2015	2016	2017	2018	2019	p-value
Mortality rate, %											
Overall	0.4	0.4	0.4	0.2	0.3	0.4	0.8	0.9	0.3	0.8	0.605
Mean LOS, days											
Overall	4.1	4.1	4.3	4.4	4.3	4.8	4.6	4.6	4.5	4.3	
Mean THC, US$											
Overall	34304	37960	39426	45253	48414	50650	55529	57599	58255	55590	

## Discussion

The pathogenesis of EoE, though incompletely understood, is thought to result from an interplay between the environment and host immune system. Antigen proteins, typically derived from food can trigger the adaptive T helper 2 (Th2) cells to produce interleukin-5 and interleukin-13, which eventually lead to the recruitment of eosinophils from peripheral blood into the esophageal tissue. This has been proposed to be the underlying pathophysiological mechanism of EoE in genetically predisposed individuals [1,2]. The role of an allergic response being a major part of EoE pathogenesis is further supported by the finding of atopy in approximately 75% of patients with EoE and other eosinophilic gastrointestinal disorders [3,12,13].

The first cases of probable EoE were reported in the late 1960s and 1970s and have since been reported mainly in developed countries [14,15]. We noted a 134% increase in the prevalence of EoE hospitalizations over the decade studied. The rising prevalence and incidence of EoE have been confirmed in many population-based studies worldwide [16-18]. Despite the rising incidence, EoE is still considered a rare disease. Our findings suggest an average incidence rate of 11.2 cases per 100,000 adults over the decade, which was similar to the findings of Eke et al. on EoE hospitalizations in the US [19]. This almost parallels that of inflammatory bowel disease which is approximately 16 per 100,000 persons [20].

A male predominance was confirmed in this large national database study, and patients aged 18-44 years were the most affected. These findings are consistent with previous literature on EoE [16-18]. Within the study period, we did notice an increase in the incidence of EoE in the elderly population which is consistent with the natural course of this chronic progressive disease. Patients with EoE were three times more likely to be Caucasian than other racial groups, as shown in multiple studies [21,22]. The reason for this racial and gender difference is unknown and little is known about the different clinical presentations, if any, amongst EoE patients with different genders and racial backgrounds.

Several mechanisms have been proposed to explain the rising incidence and prevalence of EoE over the past years. The widespread use of endoscopy with biopsies as well as increased awareness amongst physicians has been proposed as a reason for the increased detection; however, this alone is unlikely to explain the meteoric rise in the incidence of EoE due to the reasons discussed below.

In a study done by de Rooij et al., they noticed a 2.6-fold rise in endoscopies with biopsies compared to a 316-fold increase in EoE incidence over their study period suggesting other factors may be responsible for the increased incidence [18]. In addition, data extrapolated from the rising incidence of other atopic diseases parallels that of EoE giving more credence to the role of environmental allergens in the pathogenesis of EoE [23,24]. It has also been suggested that modern hygienic practices in childhood may have played a part in the observed increase in EoE incidence due to less exposure to microbes, which subsequently leads to increased sensitivity to microbes/allergens in adulthood [25]. The increased use of proton pump inhibitors over the years has also been suggested to have a role in the increased EoE incidence mainly due to its acid-suppressing effect by reducing peptic digestion of food allergens and microbes increasing exposure of these allergens/microbes to the gastrointestinal mucosa [18,26,27]. Overall, there is evidence supporting the pathogenic role of allergens, but the exact role of these environmental allergens remains unclear in the etiopathogenesis of EoE.

Our study highlighted the difference in EoE distribution by hospital region as well. Most of the hospitalized patients with EoE were from the South and Midwest regions of the United States. This finding was similar to what was found in a study on EoE prevalence by Dellon et al. using health insurance claims from a large database in the US [28]. This observation may be due to the different environmental exposures in the various regions of the US. About 95% of patients in this study were treated in the urban setting which again supports the theory proposed earlier that the environment plays a major role in the pathogenesis of EoE.

This study has some limitations. Firstly, the NIS reports data on hospitalizations, not individual patients; patients with recurrent hospitalizations may count as multiple admissions, affecting the prevalence rate. Secondly, this study is observational, and our findings cannot establish causation. Finally, since the NIS is an administrative database, ICD codes used to confirm diagnoses may not have wholly matched clinical parameters. Despite the potential limitations, this was a comprehensive study using a nationally representative sample, allowing us to provide insights into the hospitalization trends of EoE to statistically significant proportions.

## Conclusions

The prevalence rate of hospitalized patients with EoE is on the rise and is most prevalent amongst the young population, male gender, and Caucasians. The average prevalence rate among hospitalized patients with EoE over the decade in review almost parallels that of inflammatory bowel disease, representing a significant burden of disease for a condition that has only been recognized over the past three decades. This increasing prevalence likely represents the result of a combination of improved diagnostic accuracy leading to increased detection and an increase in the incidence of atopic disease associated with this EoE. However, further research is needed to fully understand the underlying factors driving these observed trends.
